# Abnormal Gut Microbiota is Associated with Depressive-like Behavior in the Rat Model of Autoimmune Prostatitis

**DOI:** 10.1007/s12010-025-05294-1

**Published:** 2025-06-27

**Authors:** Yihan Wang, Xin Zhu, Yandong He, Wenlong Lu, Zhong Wang, Feng Liu

**Affiliations:** Department of Urology, Shanghai Fengxian District Central Hospital, Urology Specialty Alliance of Fengxian District, No.6600, Nanfeng Road, Shanghai, 201499 P.R. China

**Keywords:** Prostatitis, Depression, Gut microbiota dysbiosis, Autoimmunity

## Abstract

Many patients with chronic prostatitis (CP)/chronic pelvic pain syndrome (CPPS) also experience depression, although the pathogenesis was unclear. Some studies have shown that gut microbiota disorder might be related to both prostatitis and depression. Therefore, we investigated the alteration of the gut microbiota in autoimmune CP/CPPS complicated with depression in a rat model. The rat model of autoimmune CP with comorbid depressive-like behavior was constructed by experimental autoimmune prostatitis (EAP) and chronic unpredictable mild stress (CUMS). Then, enzyme linked immunosorbent assay (ELISA) was performed to determine the concentration of inflammatory cytokines. The depression-like behaviors in rats were also detected using the open field test (OFT), forced swimming test (FST), and sucrose preference test (SPT). The composition of the gut microbiota and the microbial profile in rats were measured by bioinformatics analysis of the 16S rRNA gene sequence. The level of interleukin (IL)-1β, IL-6, tumor necrosis factor α (TNF-α), and depression-like behavior in rats showed that the CP/CPPS complicated with depression model was established successfully. The results of the 16S rRNA sequence analysis showed disordered abundance, uniformity, and composition of gut microbiota in EAP + CUMS rats. Over 60 gut microbial metabolic pathways were predicted to be disordered among the four groups of rats. Additionally, metabolomics profiling indicated significant differences in EAP + CUMS rats compared to other groups, including reduced PWY-922, PWY-5198 and PWY-7159, and increased PWY0-321 and PWY-6629, which were predicted to be altered by *Lactobacillus, Shigella, Blautia, Clostridiales, Comamonadaceae* and *Rhodospirillales*. Our results have shown that gut microbiota disorders are associated with depressive-like behavior in experimental prostatitis. Our findings concerning gut microbiota provide new insights in understanding prostatitis-associated depression.

**Supplementary Information:**

The online version contains supplementary material available at 10.1007/s12010-025-05294-1.

## Introduction

Prostatitis was a genitourinary disease that affected adult men and can increase the risk of prostate cancer [1, 2]. As classified by the National Institutes of Health, prostatitis can be divided into acute bacterial prostatitis (type I), chronic bacterial prostatitis (type II), chronic prostatitis (CP)/pelvic pain syndrome (CPPS) (type III), and asymptomatic inflammatory prostatitis (type IV) [3]. Among these, CP/CPPS was the most common type of prostatitis accounting for 90–95% of all diagnoses [4]. In addition to pain, the clinical symptoms of CP/CPPS were associated with lower urinary tract disease and sexual and intestinal dysfunction, accompanied by mental and psychological disorders, such as anxiety and depression, which can adversely affect the quality of life of patients [5–8].

Many studies have shown that depression was prevalent among prostatitis patients [9]. Approximately 17% of these patients experienced depression, and severely affected patients may even be at risk of suicide [10]. Although the etiology of CP/CPPS was unclear, microbial infection and autoimmune factors were closely related to the disease [10]. The gut microbiota was involved in the induction of chronic inflammatory environment [11]. Dysbiosis in the gut microbiota had been shown to promote the progression of prostate cancer in antibiotic exposure mice via the Toll-like receptor 4 (TLR4) activated transcription factor-κB (NF-κB)—interleukin (IL)−6- signal transducer and activator of transcription 3 (STAT3) axis [12]. Furthermore, the impact of intestinal microbiota on estrogens could also contribute to the pathogenesis of prostate cancer [13]. However, certain microbes had been found to have an anti-tumor role by triggering a strong immune response [14].

The gut microbiota played a key role in influencing the occurrence and development of depression by regulating the interaction between the gut-brain axis and the host [15, 16]. The severity of depression can be reduced by transplanting the gut microbiota of healthy donors [17, 18]. Therefore, the disordered composition of the gut microbiota was a potential pathogenic factor for depression. The dysfunction in the prostate and changes in the gut microbiota in CP/CPPS patients might be related to depression caused by CP/CPPS [17]. Additionally, autoimmune reactions can contribute to the induction and progression of CP/CPPS, potentially exacerbating depressive symptoms [3, 19]. Maintaining a stable composition of the gut microbiota was important for balancing of integrity and inflammation [20, 21]. However, it was not known how changes in the gut microbiota underlie depressive-like behavior in the context of CP/CPPS.

To determine the pathology, we first established a rat model of experimental autoimmune CP (EAP) with comorbid depressive-like behavior, which was a classic model of CP/CPPS. We conducted behavioral tests and analyzed inflammatory factors in rats. Then, the changes in the gut microbial community and related signaling pathways induced by EAP through 16S rRNA sequencing. Additionally, we also examined the differences in metabolic pathways in rats with EAP/depression.

## Methods

### Animal

**Fig. 1:**
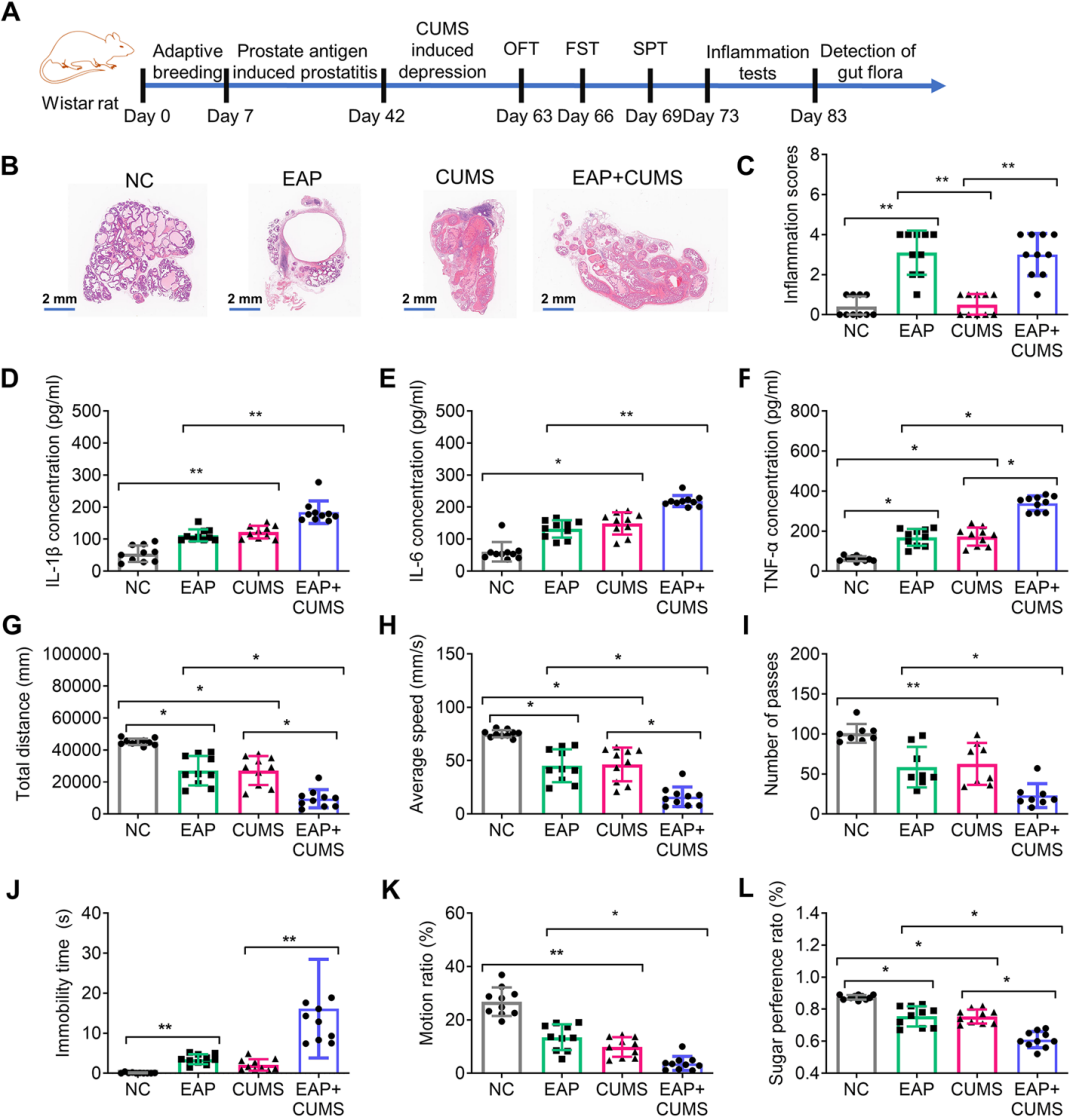
EAP and depression model in rats. (**A**) The experimental paradigm. (**B**) Images of prostate tissue after H&E staining. (**C**) Quantification of the inflammation level in prostate tissue. (**D-F**) ELISA was performed to estimate the levels of IL-1β (**D**), IL-6 (**E**), and TNF-α (**F**) in rats. (**G-I**) The performance of the three groups of rats in OFT, including **G**: Total distance in OFT, **H**: Average speed, and **I**: The number of passes. (**J**) The immobility time of rats in FST. (**K**)The motion ratio of rats in FST. (**L**) The sugar performance ratio of rats in SPT; ^NS^ P > 0.05, * P < 0.05, and ** P < 0.01

In total, 60 male Wistar rats (8–16 weeks old) were purchased from Shanghai Slack Laboratory Animal Co., Ltd [SCXK (Shanghai) 2022–0004]. The animal study was approved by the Institutional Animal Care and Use Committee of Shanghai Chengxi Biotechnology Co., Ltd (Approval NO.: CX052412030). All specific pathogen-free (SPF) rats were fed at the animal center of Shanghai Chengxi Biotechnology Co., Ltd and acclimatized to the environment for one week. The temperature was maintained at 22 ± 0.5 °C with a 12 h light and 12 h dark cycle. The rats had access to food and water ad libitum. These rats were evenly divided into four groups: the negative control group (NC), the experimental autoimmune prostatitis (EAP) group, the chronic unpredictable mild stress (CUMS) group, and the EAP + chronic unpredictable mild stress (CUMS) group. The Institutional Animal Care and Use Committee of Shanghai Jiao Tong University approved the animal experiments and strictly followed the recommendations. The experimental design for the study was shown in Fig. 1A. The list of reagents used in the study was shown in supplement Table S1.

### Purification of Prostate Steroid-Binding Protein (PSBP)

We purified PSBP using the method described in another study [22]. Briefly, the prostate tissue was lysed in lysis buffer containing 0.09% NaCl, 0.5% Triton X-100, and protease inhibitor mixture. The lysate was then centrifuged for 20 min at 4 °C and 12,000 g/min. The supernatant was collected, and the concentration was determined using a BCA kit (cat. ab102536, Abcam, England). PSBP was concentrated to 40 mg/mL and stored at −80 °C for further analysis.

### Experimental Autoimmune Prostatitis (EAP) Model

Experimental autoimmune prostatitis (EAP) was used to construct the animal model of CP/CPPS [23, 24]. In this study, we established an EAP rat model following a previously reported procedure. PBSP was emulsified with complete Freund's adjuvant (Sigma-Aldrich) at a 1:1 ratio. The emulsified PBSP and PBSP were administered to the left prostate (0.1 mL) and the abdominal cavity (0.1 mL) of EAP and EAP + CUMS rats, respectively. In contrast, rats in NC and CUMS groups received 0.9% saline instead of PBSP. Additionally, four weeks later, the rats with EAP and EAP-CUMS were immunized with PBSP emulsified with incomplete Freund's adjuvant. To prevent infection, all rats were injected with penicillin for seven days after the operation.

### Chronic Unpredictable Mild Stress (CUMS) Procedure

The model of depression induced by CUMS was established following a previously described method [25]. The rats were allowed to acclimate to the environment for a week before the CUMS experiment. After acclimation, the rats in the NC group remained undisturbed in their cages in a separate room, while rats in other groups were exposed to a stressor at different time points every day. Rats were exposed to one or two different stressors daily for 42 days. The stressors included (1) fasting and water fasting for 12 h, (2) night flash 120 times/min for 6 h, (3) swimming for 20 min, (4) cage tilt (45°) for 12 h, (5) wet cage for 12 h, (6) reversal of the 12 h/12 h light/dark cycle, (7) body restraint for 3 h, (8) transforming light and dark, 2 h as a cycle, (9) electrical stimulation for 3 min, and (10) noise for 30 min (80 decibels). All stressors were administered randomly to ensure experimental unpredictability.

### Hematoxylin and Eosin (H&E) Staining

The H&E staining of prostate tissues was performed following a previously described method [26]. Briefly, the tissues were fixed in 4% paraformaldehyde (PFA) at 4 °C overnight. Next, the tissues were embedded in paraffin and sliced into thin Sects. (5 µm thick). Following deparaffinization, the slices were stained with hematoxylin solution for 5 min, soaked in HCl-ethanol five times, and rinsed with water. The slices were stained with eosin for 3 min and then washed in H_2_O, dehydrated in graded alcohol, and cleared in xylene. Finally, the slices were mounted with neutral balsam. Images were captured using a light microscope and analyzed using Image J (National Institutes of Health, Bethesda, MD, USA).

### Enzyme-linked Immunosorbent Assay (ELISA)

The supernatant of the prostate tissue was collected after centrifugation at 800 g for 20 min at 4 °C. The levels of tumor necrosis factor (TNF)-α (cat. F16960), IL-6 (cat. F3743), and IL-1β (cat. F3739) were estimated using enzyme linked immunosorbent assay (ELISA) kits (Shanghai Xitang Biotechnology Co., Ltd, Shanghai, China).

### Behavioral Tests

The motivational behavior of the rats was evaluated through behavioral tests. All experiments were carried out in a quiet environment. After exposure to CUMS, the rats were adapted to the environment for 2 h before the test. The behavior of the rats was evaluated by two tests. The rats were subjected to behavioral test at 8:00 to 16:00 after exposure to CUMS. The order of the tests was as follows: open field test (OFT), forced swimming test (FST), and sucrose preference test (SPT). Each rat was given a one week break before undergoing the next test.

### Open Field Test (OFT)

The OFT was performed to assess the exploratory activity of the rats, as described in another study [27]. The rats were positioned at the center of an open field (100 × 100 × 40 cm) and recorded for 6 min. The movement of the rats was recorded by the SMART3.0 video tracking system (Harvard Apparatus, USA). The cross number, movement distance, and speed of each rat were analyzed. The equipment was cleaned with 75% ethanol between each trial.

### Forced Swimming Test (FST)

To analyze depressive-like behavior in rats, FST was performed [28]. A clear glass cylinder (30 cm diameter, 50 cm height, and 35 cm water depth) was used for the FST. The rats were placed alone in a cylinder the day before the experiment was conducted for 15 min and returned to their cages. The water temperature was kept at 25℃. During the test, the activity of the rats in the cylinder was recorded for 6 min. The time of immobility and struggling in the water was analyzed. The water was replaced between trials. For these rats, immobility was defined as floating in the water without struggling.

### Sucrose Preference Test (SPT)

The rats were challenged in the SPT to detect depression-like behavior [29]. The rats were exposed to two bottles of water followed by two bottles of 1% sucrose for 48 h. Then, they were kept under fasting conditions, without water and food for 24 h. Next, they were administered one bottle of water and one bottle of 1% sucrose for 2 h in the dark. Then, the rats were provided the two fluids for another 2 h after changing the position of the bottles. The difference in weight between the water and sucrose bottles was measured before and after the experiment. The formula for calculating sucrose preference was as follows: sucrose consumption/total consumption of sucrose and water.

### Analysis of the 16S rRNA of the Gut Microbiota

The 16S rRNA analysis was performed by Shanghai Paisennuo Biotechnology Co., Ltd (Shanghai, China). DNA samples from fecal pellet were extracted using a DNA Extraction kit (cat. K1820001, Thermo, USA) following the provided instructions. Using the universal primers of the V4 region of bacterial 16S rRNA gene on the Illumina MiSeq platform, genomic DNA was amplified in a 20 µL reaction system. The purified DNA was analyzed via high-throughput sequencing.

The sequencing data were sent to Shanghai Paisennuo Biotechnology Co., Ltd (Shanghai, China) for processing and analysis. The sequences were further denoised, removed, and clustered by the QIIME software. Then, an operation classification unit (OTU) was generated. Based on the distribution and the results of the analysis of ASV/OTUS, the levels of microbial diversity, α-diversity, and β-diversity were analyzed. The principal coordinates analysis and 3D visualization analysis of BrayCurtis distance were performed using the R software (version 4.2.2; https://cran.r-project.org/). Kruskal–Wallis and Wilcoxon tests were performed to assess the effects of bacteria. The observed species and Chaol index and Shannon and Simpson indices were used as the indicators of species richness and community diversity, respectively. The species complexity among different samples was estimated by analyzing the β-diversity. The QIIME was used to calculate the β-diversity based on weighted UniFrac. The unweighted pair group method was used for conducting hierarchical clustering analysis. The difference in the gut microbiota among groups was analyzed by linear discriminant analysis (LDA, Score & gt; 3.19) using the Lefse software. The ASV/OTUS with less than 10 sequences and less than five samples were screened and removed, and then, nested hierarchical cross-test and random forest analysis were performed using the “classify-samples-ncv” function. The sparcc algorithm, random matrix theory, and iGraph were used for constructing the correlation matrix, determining the filtering threshold of the correlation value, and constructing related network data, respectively.

The abundance data for metabolic pathways were obtained from the Kyoto Encyclopedia of Genes and Genomes (KEGG) database, MetaCyc data, and homologous cluster (COG) data for pathway enrichment analysis. Metonomeseq and hierarchical methods were utilized for data analysis to ascertain the variances in metabolic pathways and species composition among groups.

### Statistical Analysis

The SPSS 25.0 (IBM Corp., Armonk, NY, USA) software was used for statistical analysis. The normality of distribution was evaluated using the Shapiro–Wilk test. The data that display normal distribution were tested by one-way ANOVA test. The data that did not display normal distribution were tested by Kruskal–Wallis H test. Statistical significance was defined as P < 0.05 for all group comparisons. More than three independent biological repeats were performed for each experiment unless stated otherwise.

## Results

### Rat Model of EAP Complicated with Depression

Based on the methods described in other studies, the rat model was established using PBSP-induced EAP and CUMS-induced depressive-like behavior, respectively [23, 25]. The experimental procedure was shown in Fig. 1A. To determine the effects of PBSP and CUMS on rat prostate tissue, we analyzed the histological changes in the prostate tissue by H&E staining. The prostatic epithelium and stroma showed signs of hyperemia and edema, accompanied by inflammatory cell infiltration in EAP, and EAP + CUMS groups but not in NC and CUMS groups (Figs. 1B and C). Inflammatory factors were known to affect the pathological process of prostatitis [30, 31]. To assess the effect of inflammation on rats with EAP and CUMS, the levels of IL-1β, IL-6, and TNF-α in the prostate tissue of the rats were evaluated by ELISA. In Fig. 1D-F, the results showed that IL-1β, IL-6 and TNF-α were significantly higher in CUMS and EAP + CUMS groups than NC group (all P < 0.05). The levels of IL-1β, and IL-6 were increased in EAP + CUMS than in EAP groups. The TNF-α levels were also dramatically enhanced in EAP group compared with the NC group. There was also a significantly increased of TNF-α in EAP + CUMS group compared with CUMS group. However, the difference in IL-1β, and IL-6 levels between the NC and EAP groups of rats were not significant. The highest levels of IL-1β, IL-6 and TNF-α were observed in the EAP + CUMS group. The depressive-like behaviors of rats were measured using OFT, FST, and SPT. In the OFT, the analysis of total distance, average speed and number of passes were significantly lower in the CUMS and EAP + CUMS rats than in NC rats (all P < 0.05). And EAP group also had the difference based on the total distance, and average speed compared with the NC group. EAP + CUMS had a lowest total distance (Figs. 1G-1I). In the FST, the immobility time for rats in EAP and EAP + CUMS groups was significantly longer than that for rats in the NC and CUMS groups (P < 0.01, Fig. 1J). However, the motion ratio was lower in the CUMS and EAP + CUMS groups than in the NC and EAP groups (Fig. 1K). Additionally, the sucrose preference ratio in the rats from the EAP, CUMS and EAP + CUMS groups was significantly lower than that in the NC rats, and it was the lowest in the EAP + CUMS group (Fig. 1L).

### PBSP and CUMS Exposure Induced Gut Microbial Dysbiosis in Rats

**Fig. 2:**
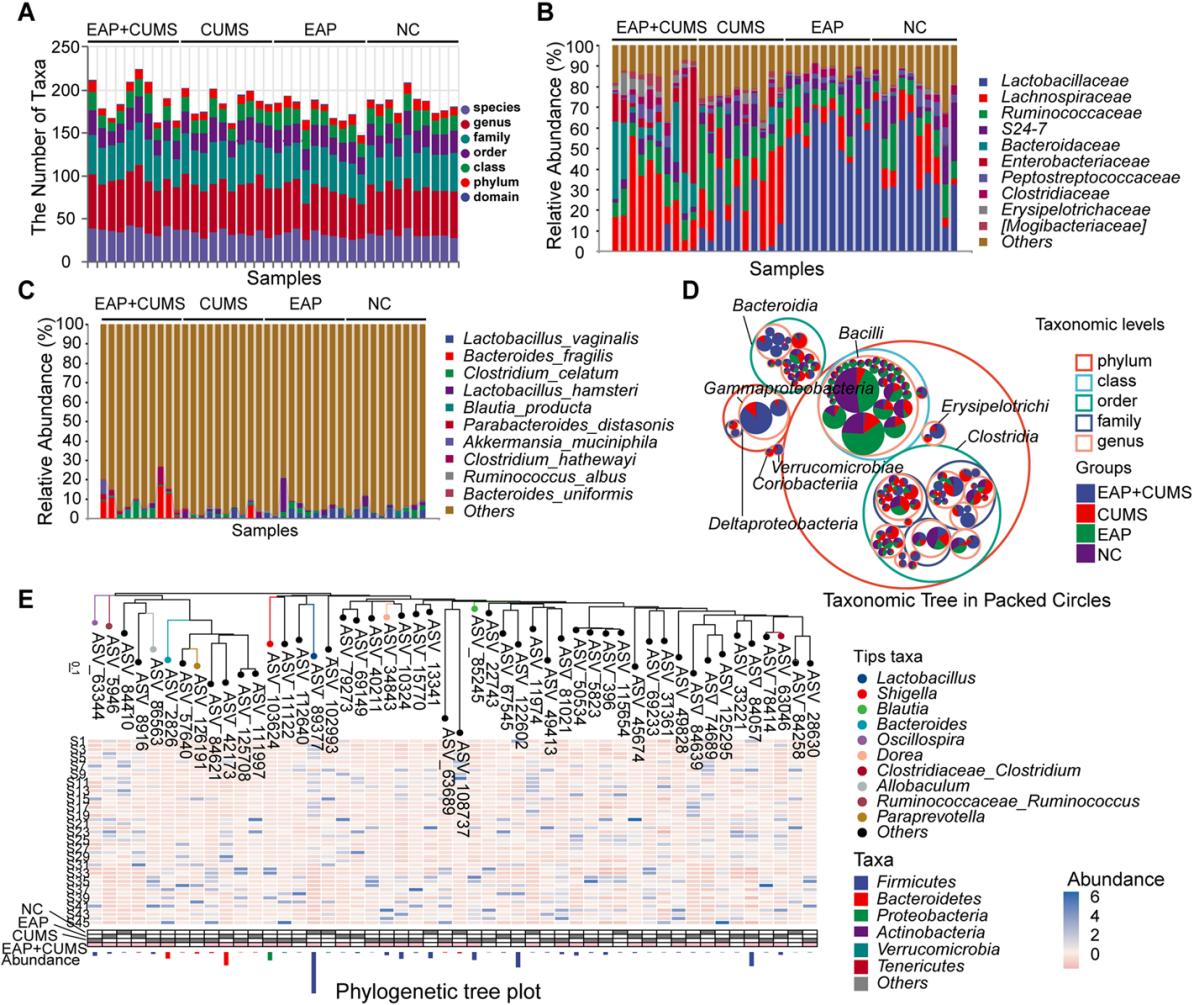
PBSP and CUMS induced gut microbial dysbiosis in rats. (**A**) The number of taxa of gut microbiota at the species, genus, family, class, phylum, and domain levels in the four groups of rats. (**B-C**) The relative abundance of gut microbiota at different taxonomic levels in the four groups; **B**: Family level; **C**: Genus level. (**D**) A taxonomic tree in packed circles for the gut microbiota at different levels of taxa in the four groups. The largest circle represents the gate level, and the shrinking circles represent classes, orders, families, genera, and species according to the gradient; different taxonomic levels are differentiated using different colors. (**E**) The phylogenetic tree shows the composition, abundance, and taxonomy of gut microbiota in different groups of rats

After establishing the EAP with depression model in rats, we investigated the alteration of gut microbiota in each group by performing 16S rRNA gene sequencing and bioinformatics analysis. As shown in Fig. 2A, the number of taxa at the species, genus, family, class, phylum, and domain levels was evaluated. The relative abundance of gut microbiota was very different among the four groups. At the family level (Fig. 2B), Lactobacillaceae was markedly reduced in CUMS group, and more seriously reduced in EAP + CUMS group; Clostridiaceae, Lachnospiraceae, Bacteroidaceae, Enterobacteriaceae, Erysipelotrichaceae, and Mogibacteriaceae were increased in CUMS group, and more significantly increased in EAP + CUMS group. At the genus level (Fig. 2C), compared to the rats of NC and EAP groups, the CUMS and EAP + CUMS group rats had a significantly lower abundance of Lactobacillus; in contrast, the abundance of Blautia, Bacteroides, Shigella, Oscillospira, Dorea, and Allobaculum were significantly higher in CUMS and EAP + CUMS groups, most of which were more significantly increased in EAP + CUMS rats. As shown in Figs. 2D and E, significant differences were found in the relative abundances of Bacterodetes, Tenericutes, and Firmicutes at phylum level; Clostridia, Bacilli, Bacteroidia, Gammaproteobacteria, and Erysipelotrichi at class level; and Lactobacillus, Bacteroides, Dorea, Shigella, and Oscillospira at genus level among the four groups. These results indicated that EAP and depression induced the dysbiosis of gut microbiota.

### Effect of CUMS on the α- and β-Diversity of Gut Microbiota in EAP Rats

**Fig. 3:**
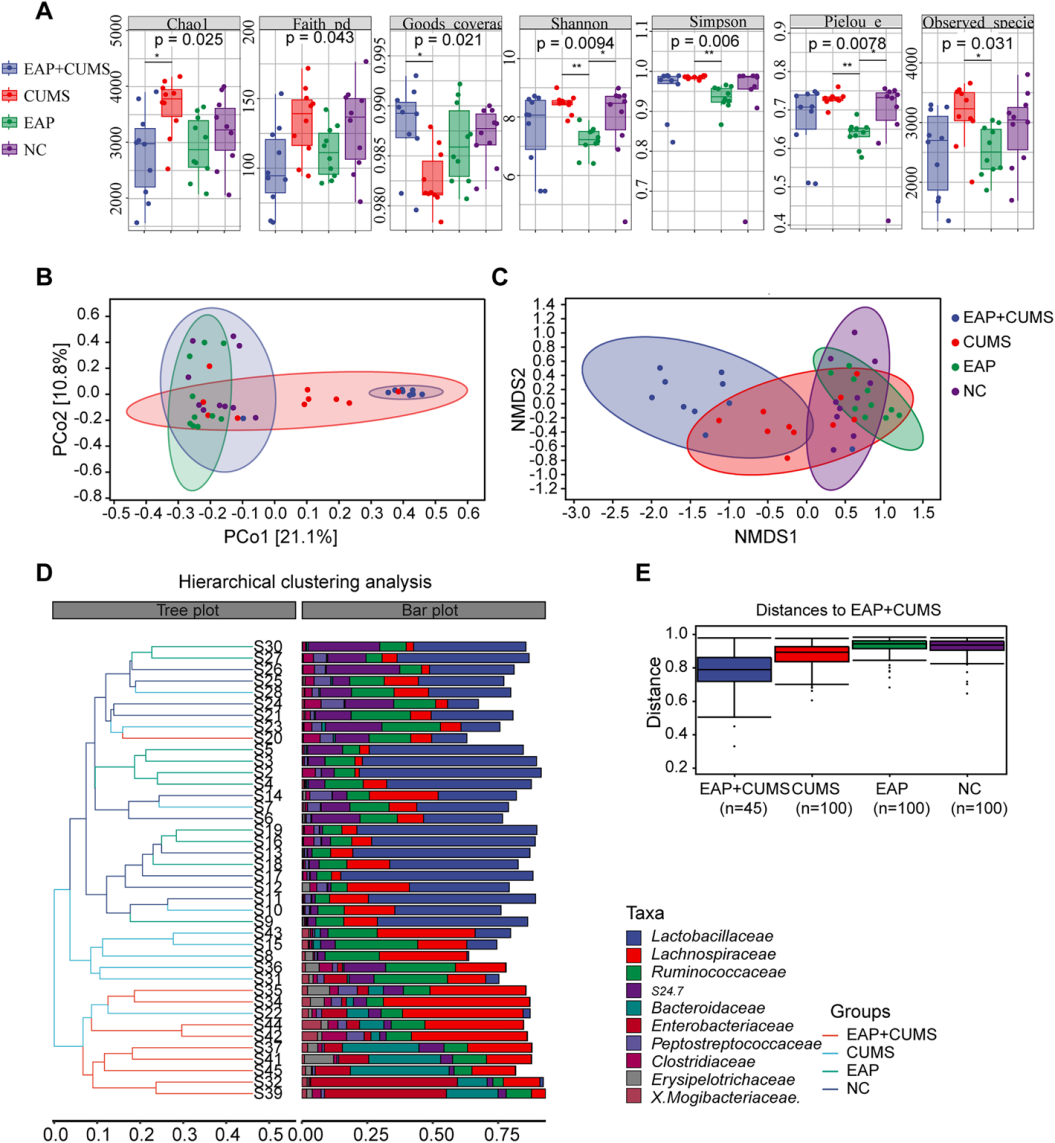
The effect of CUMS on α- and β-diversity of the gut microbiota in rats with EAP. (**A**) The α-diversity of the gut microbiota based on Chao1, Observed-species, Shannon, Simpson, Faith’s PD, Pielou-e, and Goods-coverage analysis. The β-diversity of the gut microbiota based on (**B**) PCoA (Jaccard and Bray–Curtis analysis), (**C**) NMS analysis, (D) hierarchical clustering analysis, and (**E**) permutational multivariate analysis of variance; n, the species number of gut microbiota. significant difference at P ≤ 0.05

To further elucidate the microbiota associated with the development of depression in EAP, we compared the microbial diversity among the four groups. The α-diversity and β-diversity of the gut microbiota were measured to evaluate the composition within or between different groups [32, 33]. The analysis of α-diversity based on Chao1 and Goods-coverage showed that there was no significantly different among NC, EAP, and CUMS groups, but there was a dramatically decreased of Chao1 and increased of Goods-coverage in EAP + CUMS compared with CUMS groups. For Faith-pd, no significant difference was observed among four groups. There were significant enhance of α-diversity by Shannon and Pielou-e in NC and CUMS group compared with EAP group. And the results of Simpson and Observed-species showed that there was significantly increased α-diversity in CUMS group compared to EAP group (Fig. 3A). However, for β-diversity, the PCoA plot showed that the dots in CUMS and EAP + CUMS rats were far from those in NC and EAP rats, but similar results were found in NC and EAP rats (Fig. 3B). Meanwhile, the dots of EAP + CUMS stayed away NC, EAP and CUMS groups based on the NMDS analysis (Fig. 3C). The results of the hierarchical clustering analysis at the family level showed that Lactobacillaceae, Ruminococcu, and S24.7 were present in NC, EAP and CUMS rats, but Lachnospiraceae, Bacteroidaceae, Enterobacteriaceae, Erysipelotrichacea and X.Mogibacteriaceae were mainly present in EAP + CUMS rats (Fig. 3D). Also, EAP + CUMS and CUMS group showed inter-group difference with EAP and NC group, as determined by permutational multivariate analysis (Fig. 3E). These results indicated that the composition of the gut microbiota in rats with EAP and depression was different from that in the NC and EAP, and CUMS rats.

### Comparisons of Different Species and Marker Species

**Fig. 4:**
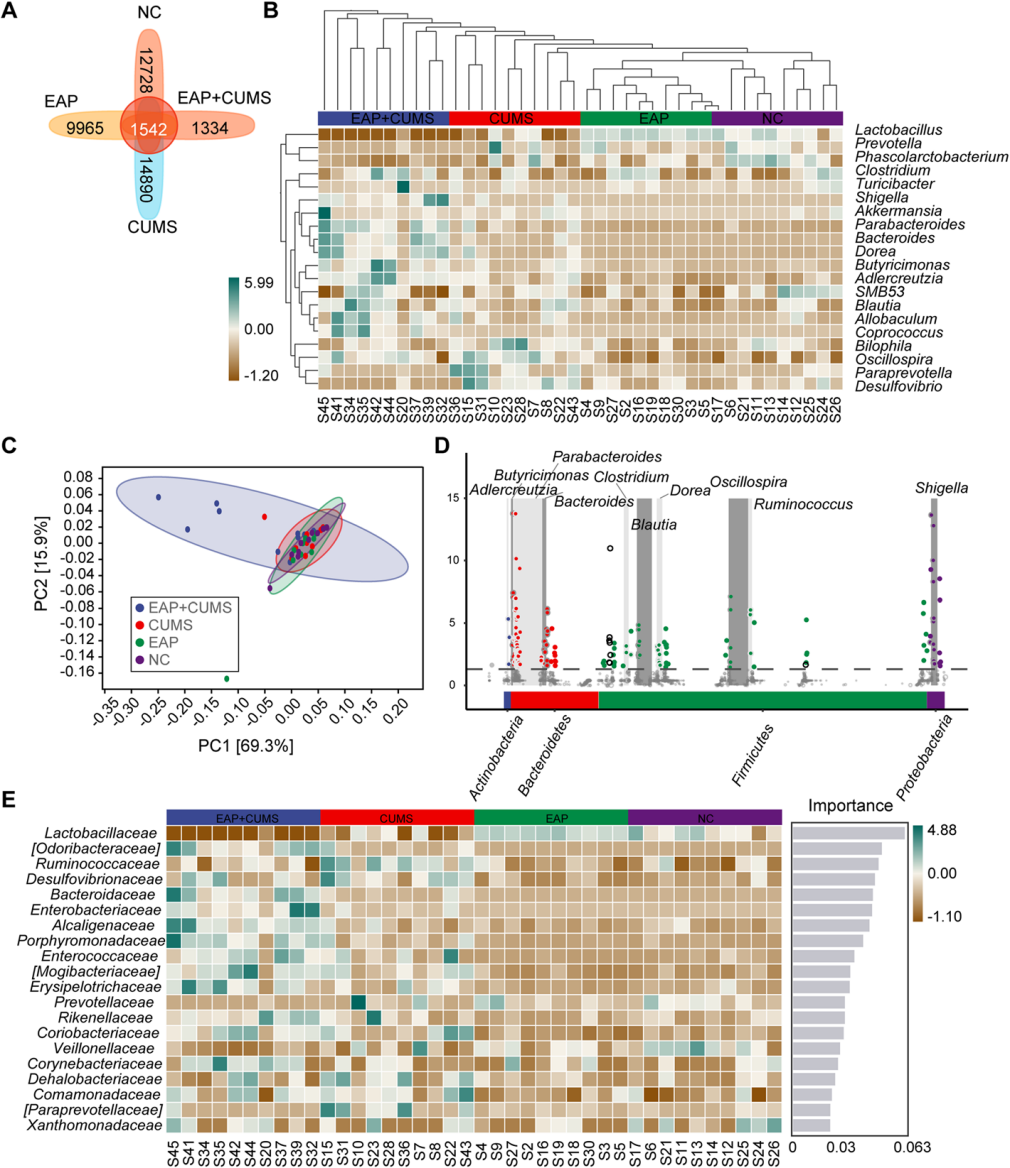
Comparisons of different species and marker species. (**A**) Venn diagram analysis for the number of gut microbiota in the four groups of rats. (**B-C**) Analysis of species composition of the gut microbiota among the four groups. **B**: Heat map analysis; **C**: PCA analysis at the species level. (**D**). MetagenomeSeq analysis of the taxonomic distribution of the gut microbiota. The dot represents a taxon of gut microbiota, and its size represents the relative abundance. A change in the color dot indicates a significant difference in the taxa among the four groups, while gray indicates no significant difference. (**E**) Random forest analyses of the marker species of the gut microbiota at the family level

**Fig. 5:**
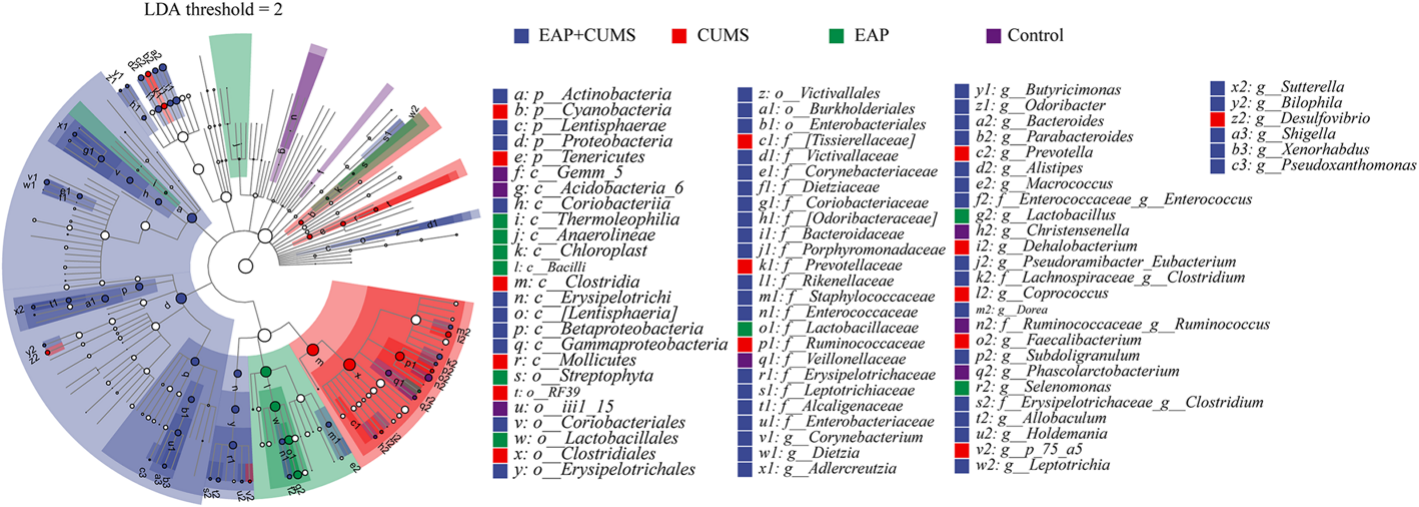
The EAP + CUMS highly correlated gut microbiota among the four groups of rats was determined by LDA and LEfSe analysis. The hierarchical relationship of gut microbiota from phylum to genus is shown in the branching diagram. The size of the circle indicates the average relative abundance of the taxon; hollow circles indicate that the abundance of the taxon is not significantly different among groups, and circles of different colors (such as blue and green) indicate a high abundance of the taxon and significant differences among groups

Marker species referred to microbial taxa (e.g., species, genera, or OTUs) identified through statistical or machine learning methods that can significantly differentiate between specific groups (disease, health, or different treatment groups.). In microbiome studies of prostatitis, bacterial genera such as Lactobacillus, Corynebacterium, Bacteroides, and Prevotella had been identified as marker species distinguishing CP/CPPS from healthy controls [34–36]. In our study, the marker species, which could distinguish the specific microbial taxa among NC, EAP, CUMS and EAP + CUMS groups, were analyzed. As shown in the Venn diagram, there were 1542 gut microbes in all groups, while 12728, 9965, 14,890 and 13340 different gut microbes were found in NC, EAP, CUMS and EAP + CUMS rats, respectively (Fig. 4A). A systematic cluster analysis was performed to compare the species composition among the four groups. Based on the clustering results, the species composition of the top 20 genera of gut microbiota was analyzed. The species composition of gut microbiota in EAP + CUMS group and CUMS group were differed significantly from NC and EAP (Fig. 4B). Prevotella, Phascolarctobacterium, and SMB53 in NC group. Lactobacillus in EAP group. Bilophila, Oscillospira, Paraprevotella, and Desulfovibrio in CUMS group. And Clostridium, Turicibacter, Shigella, Akkermansia, Parabacteroides, Bacteroides, Dorea, Butyricimonas, Adlercreutzia, Blautia, Allobaculum, and Coprococcus in EAP + CUMS group. The PCA plot showed that there was significant difference in EAP + CUMS group compared with other three groups (Fig. 4C). In EAP + CUMS groups, the range of PC1 was from −0.3 to 0.2. However, in NC, EAP and CUMS group, the range of PC1 was from −0.05 to 0.1. Then, the composition of the gut microbiota at the level of different taxa was analyzed by metagenomeSeq analysis. The results showed that the adjPvalues of Actinobacteria (Adlercreutzia), Bacteriodetes (Bacterides and Parabacterides), Firmicutes (*Blautia* and *Oscillospira*), and Proteobacteria (*Bilophia* and *Shigella*) were significantly different among the four groups (Fig. 4D). Additionally, the Random Forests showed the value of the species in EAP + CUMS group, and the top five species were Lactobacillaceae, Odoribacteraceae, Ruminococcaceae, Desulfovibrionaceae, and Bacteroidaceae (Fig. 4E). We also performed the EfSe analyses to determine the EAP + CUMS highly correlated microbiota among the four groups of rats. As shown in Fig. 5, when the current LDA Thershold was 2, the NC, EAP, CUMS and EAP + CUMS groups contained 7, 9, 15, and 50 highly related microbiota, respectively.

### Dysbiosis of the Gut Microbiota Profile in EAP+CUMS Rats

**Fig. 6:**
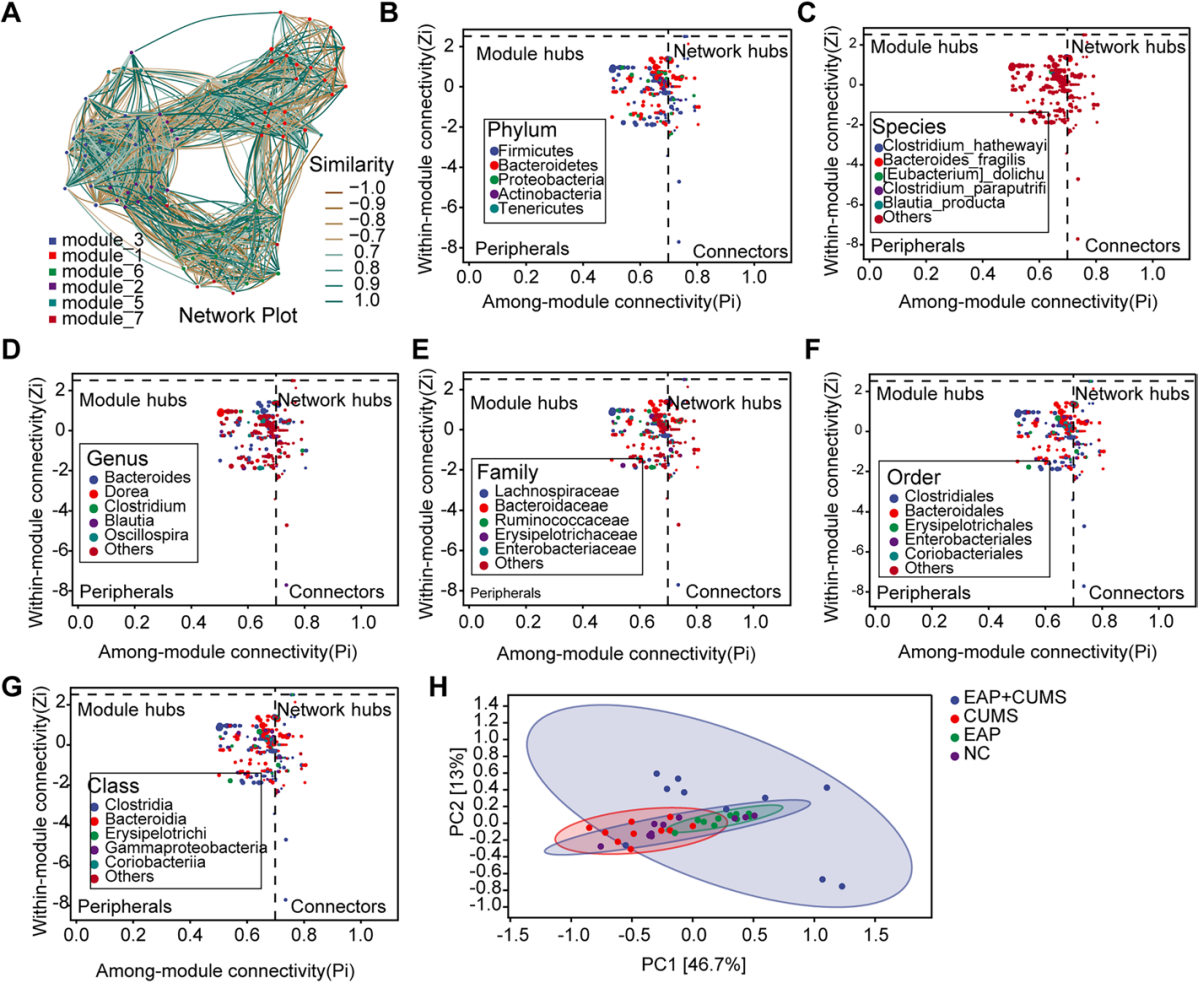
Dysbiosis of gut microbiota profile in EAP + CUMS rats. (**A**) The relationship between gut microbial members among the four groups were determined by associate network analysis. The size of the dot represents the ASV/OTUs abundance. (**B-G**) ZIPI analysis was performed to determine the hub species of the gut microbiota at the phylum level (**B**), species level **(C)**, and genus level (**D**), family level **(E)**, order level **(F)** and class level **(G)**. Peripherals, module hubs and connectors, and network hubs represented specialists, species close to generalists, and the super-generalists in the microbial network, respectively. (**H**) PCoA analysis of the functional unit (EC/KO/COG) for gut microbiota

**Fig. 7:**
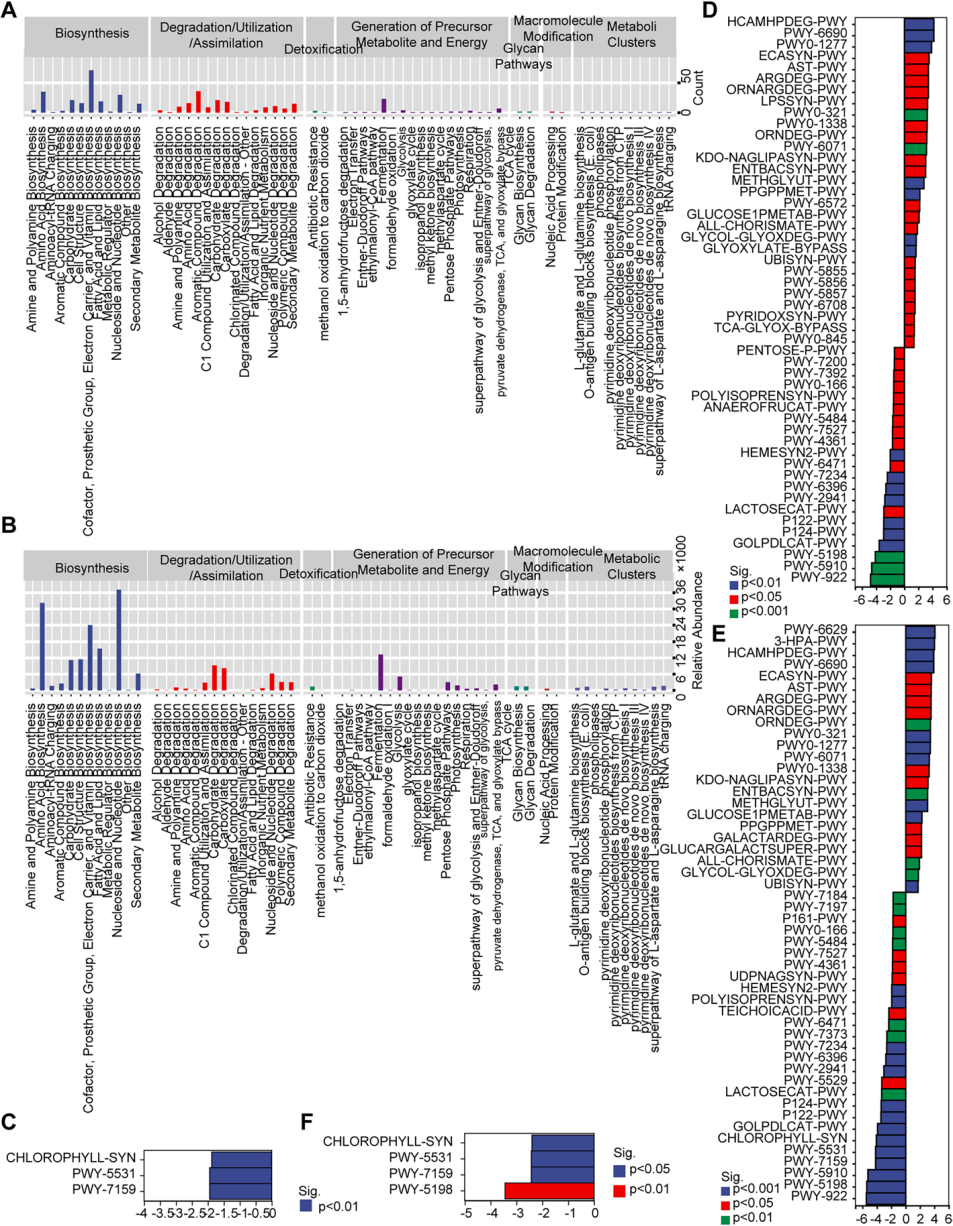
MetagenomeSeq analysis for the different metabolic pathways among four groups. (**A**) The number of different metabolic pathways in four groups; (**B**) The relative abundance of metabolic pathways in four groups. **(C-F)** The comparion of different metabolic pathways among four groups. EAP + CUMS group vs. NC group; (**C**) EAP vs CUMS groups; **(D)** EAP + CUMS vs NC; **(E)** EAP + CUMS vs EAP; **(F)** EAP + CUMS vs CUMS group

The similarity of gut microbiota in the four groups was determined by associated network analysis. The similarity between the 7 modules of gut microbiota with the most OTU in the four groups is shown in Fig. 6A. The among/within-module connectivity showed that the keystone species of gut microbiota belonged to Firmicutes, Bacteroidetes, Proteobacteria, Actinobacteria, and Tenericutes at the phylum level (Fig. 6B), Clostrium-hatgewayi, Bacterodies-fragilis, Eubacterium-doichum, Clostridium-paraputrifis, Blautia-producta at species level (Fig. 6C), Bacterodies, Dorea, Clostridium, Blautia, and Oscillospira at the genus level (Fig. 6D), Lachnospiraceae, Bacteroidaceae, Ruminococcaceae, Erysipelotrichaceae, Enterobacteriaceae at family level (Fig. 6E), Clostriales, Bacteriodales, Erysipelotrichales, Enterobacteriales, and Coriobacteriales at order level (Fig. 6F), and Clostridia, Bacteroidia, Erysipelotrichi, Gammaproteobacteria, and Coriobacteriia at class level (Fig. 6G). Next, we predicted the functional abundance based on the sequence abundance of marker genes in the four groups of rats. Comparing them directly was difficult because of the large number of functional units (EC/KO/COG). The PCoA was performed, which showed that there was a significant difference in the metabolic phenotype in EAP + CUMS rats compared to that in other three groups rats, but the difference between NC and EAP rats was not significant (Fig. 6H). More than 60 disrupted metabolic pathways were predicted among the four groups. Compared to the NC group, the EAP group showed enhanced signaling including biosynthesis pathways, PWY-56107/PWY-5198: degradation/utilization/assimilation pathways, generation of precursor metabolite and energy pathways, and inhibited PWY-7373: superpathway of demethylmenaquinol-6 biosynthesis II (Fig. 7A). Compared to the NC group, the high relative abundance of metabolic pathways in EAP + CUMS rats were biosynthesis pathways, including amino acid biosynthesis, nucleoside and nucleotide biosynthesis, and cofactor, prosthetic, group, electron, carrier, and vitamin biosynthesis (Fig. 7B). Three significantly different metabolic pathways were detected between EAP and CUMS groups (Fig. 7C). There were more than 29 enhanced metabolic pathways and 21 inhibited metabolic pathways in EAP + CUMS group compared to NC group (Fig. 7D). More than 50 metabolic pathways were obviously difference in EAP + CUMS group in contrast with EAP group, including 23 promoting metabolic pathways and 27 inhibited metabolic pathways **(**Fig. 7E). And there were four inhibited pathways in EAP + CUMS compared with CUMS group **(**Fig. 7F).

**Fig. 8:**
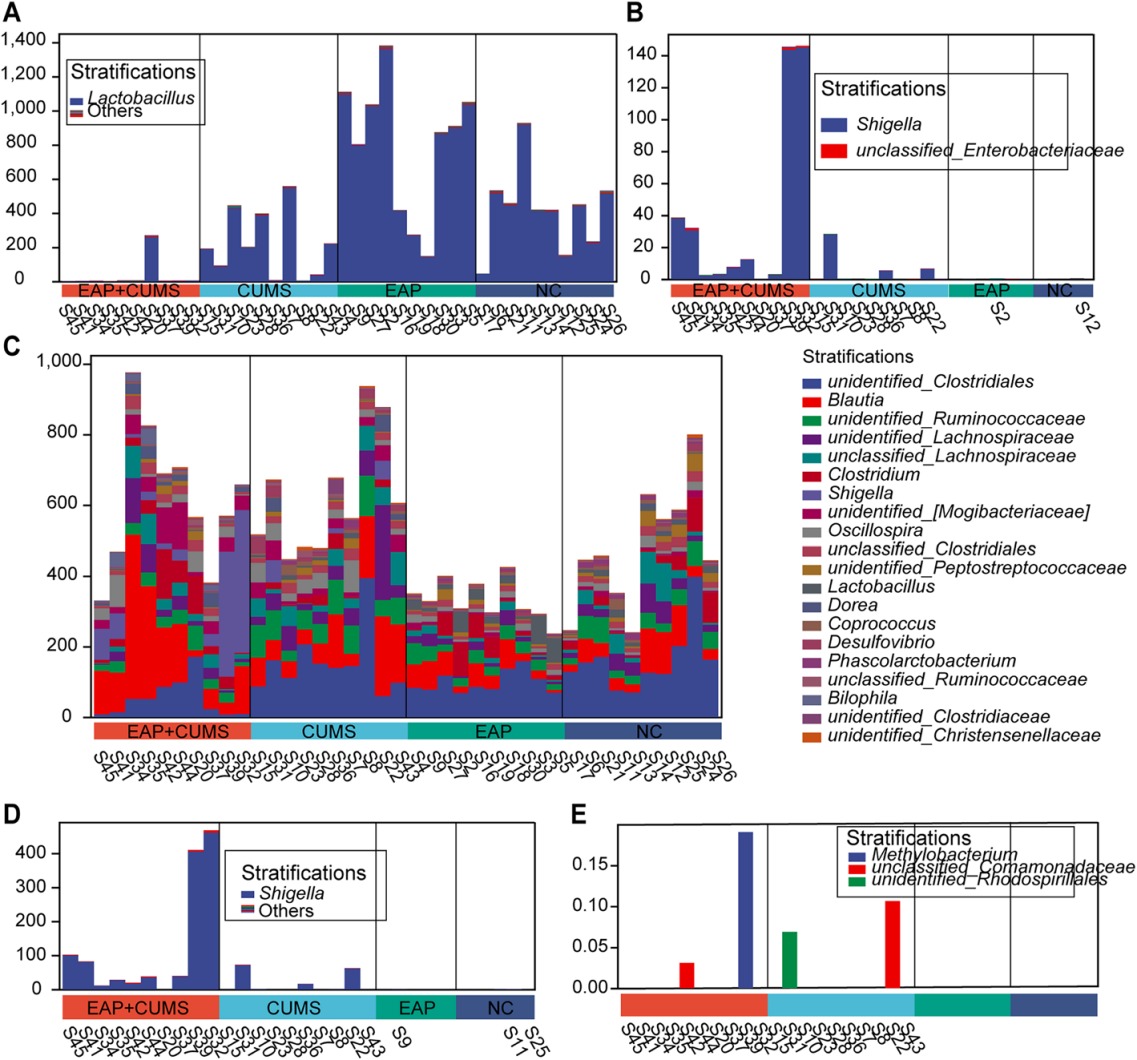
The species composition of the gut microbiota related to different metabolic pathways. The species composition of (**A**) PWY-922, (**B**)PWY0-321, and (**C**) PWY-5189; **(D)** PWY-6629; and **(E)** PWY-7159

Additionally, the species composition contributing to the five typically dysregulated metabolic pathways in EAP + CUMS was identified, including reduced PWY-922 (mevalonate pathway I) with *Lactobacillus* (Fig. 8A), increased PWY-321(phenylacetate degradation I (aerobic)) with Shigella (Fig. 8B), and reduced PWY-5198 (tetrapyrrole biosynthesis II (from glycine)) with *Blautia, Clostridiales, Ruminococcaceae, Lachnospiraceae, Lachnospiraceae*, and *Oscillospira* (Fig. 8C). There was an increase in PWY-6629 (superpathway of L-tryptophan biosynthesis) with *Shigella* (Fig. 8D), and a reduction in PWY- 7159 (chlorophyllide a biosynthesis III (aerobic, light independent)) with *Methylobacterium, Comamonadaceae,* and *Rhodospirillales* (Fig. 8E). Also, *Dorea, Coprococcus, and Desulfovibrionaceae* encoded the genes related to these metabolic pathways (Fig. 8).

## Discussion

The characteristics of gut microbiota can vary with alteration in the pathological state [37]. The multiplicity of gut microbiota in patients with CP/CPPS differed from that of healthy individuals [17]. Microbial disorders can cause the progression of prostatitis through the microbiota-gut-brain axis, which can destroy the central nervous system, leading to conditions such as PD, AD, and depression [38–40]. CP/CPPS was often complicated by depression [4]. Recent studies suggested that gut dysbiosis may contribute to the pathological progression of prostatitis through the "gut-prostate axis” [35, 41].Potential mechanisms included: systemic low-grade inflammation triggered by translocation of microbial metabolites, which exacerbates local prostatic inflammatory responses, and dysfunction of the gut-brain axis, which may promote depressive-like behaviors by modulating neurotransmitter systems such as serotonin and γ-aminobutyric acid (GABA). Notably, chronic psychological stress-induced overactivation of the hypothalamic–pituitary–adrenal (HPA) axis could further increase the release of pro-inflammatory factors (like IL-6, TNF-α), creating a vicious cycle of neuroimmune dysregulation and microbial imbalance [42, 43]. However, current evidence primarily derived from animal models or correlational studies, and the direct causal relationships among prostatitis, gut microbiota disruption, and depressive symptoms remained incompletely elucidated, particularly due to a lack of clinical longitudinal data. Therefore, further exploration of the molecular mechanisms underlying this interaction network was crucial for developing microbiota-targeted therapeutic strategies. In this study, we established EAP and depression model in rats and investigated the alterations in their gut microbiota. The levels of IL-1 β, IL-6, and TNF-α were significantly higher in CUMS and EAP + CUMS groups. The TNF-α levels were also increased in EAP group. Our results matched the findings of previous reports [30]. The data from various tests, such as OFT, FST, and SPT, indicated that CUMS induced depression-like behaviors in EAP rats. These findings validate the use of rat models for EAP, CUMS, and EAP + CUMS in the study of gut microbiota.

The gut microbiota was closely related to the depressive behavior of animals with pathological pain [44]. However, there were limited reports on whether intestinal microflora was involved in depression induced by EAP. To understand the alterations in the gut microbiota and metabolic profile in EAP and depression, 16S rRNA gene sequencing was performed to detect the evenness, richness, and composition of the gut microbiota. In our study, the diversity analyses showed significant differences in EAP + CUMS rats compared to the NC, EAP and CUMS rats. A total of 20 types of gut microbes in EAP + CUMS rats were not found in the rats from the other groups. The EAP + CUMS rats had microorganisms from more varieties of genera, including *Lactobacillus, Bacteroides, Dorea, Shigella, Paraprevotella* and *Oscillospira*. Microbiota disorders strongly affected the EAP + CUMS rats, which matched the findings of recent reports. The gut microbiota was correlated with the metabolic phenotype [45, 46]. Therefore, we also investigated the biological connection between the gut microbiota and metabolic phenotype. The relative abundance of biosynthesis, utilization, degradation, assimilation, detoxification, generation of precursor metabolite and energy, glycan pathways, macromolecule modification, and metabolic clusters were significantly different in the EAP + CUMS group, based on the marker genes. Additionally, the reduced metabolic pathways PWY-922, PWY-5198 and PWY-7159, and increased pathways PWY0-321 and PWY-6629 were associated with EAP + CUMS. The gut microbiota was closely associated with the metabolic phenotype. Abnormalities in PWY—7159 (such as glycolysis—related pathways) could reflect an imbalance in energy metabolism, which was related to activity inhibition in depressive—like behaviors. Changes in PWY—5198 (such as short—chain fatty acid synthesis pathways) were associated with the production of anti—inflammatory metabolites, which may exacerbate the inflammatory response. An increase in PWY0—321 (such as oxidative stress—related pathways) may indicate a decrease in the ability of the gut microbiota to clear toxic metabolites. Many metabolites were produced in the gut microbiota. These metabolites played an important role in maintaining intestinal dynamic balance and systemic immunity, and affected the occurrence and development of a variety of diseases [47]. In this study, we did not analyze the metabolites, we will further analyze the metabolites in the gut microbiota of CP/CPPS with depression in the next study.

Depression was largely influenced by the brain–gut–microbiota axis, as demonstrated by the fact that vagotomy blocked depression-like behavior in rodents who underwent fecal microbiota transplantation [48]. The inhibited mevalonate pathway (regulated by PWY-922) was associated with depression and alzheimer’s disease, which could be alleviated with plant polyprenols [49]. Additionally, phenyl acetate (regulated by PWY0-321) had been suggested as a diagnostic biomarker for major depression [50]. These claims were supported by our research. These findings enhanced the understanding of the role of intestinal flora in prostatitis-related depression.

Our study had some limitations. Although our study demonstrated an association between gut microbiota dysbiosis and depressive-like behaviors in the prostatitis model, the underlying biological mechanisms remain incompletely elucidated. Several critical scientific questions required further investigation: (1) The lack of quantitative measurements of key microbial metabolites (e.g., short-chain fatty acids, tryptophan derivatives) and their potential blood–brain barrier permeability prevents comprehensive understanding of gut-brain axis regulation; (2) The precise role of vagal nerve signaling in microbiota-gut-brain communication; and (3) The possible effects of localized prostatic inflammatory factors on blood–brain barrier integrity. These mechanistic questions will be prioritized in our subsequent research. Furthermore, validation through larger-scale experimental studies and clinical translational research was warranted.

In summary, we established a rat model of depression with prostatitis, and determined the disorders in gut microbiota and potentially related metabolic phenotypes. Typically, the EAP + CUMS rats showed reduced levels of PWY-922, PWY-5198 and PWY-7159, as well as increased levels of PWY0-321 and PWY-6629 compared to other groups. These disorders might be related to the pathological mechanism of prostatitis-related depression. Our findings on gut microbiota could provide new insights into understanding prostatitis-associated depression.

## Supplementary Information

Below is the link to the electronic supplementary material.

ESM 1 (DOCX 16.4 KB)

## Data Availability

The datasets obtained and analyzed during the current study were made available from the corresponding authors through request.

## References

[1] Morris, K. E., Grimberg, D., Arcot, R., & Moul, J. W. (2021). Aggressive prostate cancer masquerading as acute prostatitis*. Can J Urol, 28*(4), 10799–10801. 34378519

[2] Magri, V., Boltri, M., Cai, T., Colombo, R., Cuzzocrea, S., De Visschere, P., Giuberti, R., Granatieri, C. M., Latino, M. A., Larganà, G., Leli, C., Maierna, G., Marchese, V., Massa, E., Matteelli, A., Montanari, E., Morgia, G., Naber, K. G., Papadouli, V., Perletti, G., Rekleiti, N., Russo, G. I., Sensini, A., Stamatiou, K., Trinchieri, A., & Wagenlehner, F. M. E. (2019). Multidisciplinary approach to prostatitis*. Arch Ital Urol Androl, 90*(4), 227–248. 10.4081/aiua.2018.4.227 30655633

[3] Liu, Y., Mikrani, R., Xie, D., Wazir, J., Shrestha, S., Ullah, R., Baig, M., Ahmed, A., Srivastava, P. K., Thapa, K. B., & Zhou, X. (2020). Chronic prostatitis/chronic pelvic pain syndrome and prostate cancer: study of immune cells and cytokines*. Fundam Clin Pharmacol, 34*(2), 160–172. 10.1111/fcp.12517 31642541

[4] Wazir, J., Ullah, R., Li, S., Hossain, M. A., Diallo, M. T., Khan, F. U., Ihsan, A. U., & Zhou, X. (2019). Efficacy of acupuncture in the treatment of chronic prostatitis-chronic pelvic pain syndrome: a review of the literature*. Int Urol Nephrol, 51*(12), 2093–2106. 10.1007/s11255-019-02267-2 31468287

[5] Franco, J. V., Turk, T., Jung, J. H., Xiao, Y. T., Iakhno, S., Garrote, V., & Vietto, V. (2018). Non-pharmacological interventions for treating chronic prostatitis/chronic pelvic pain syndrome*. Cochrane Database Syst Rev, 5*(5), Cd012551. 10.1002/14651858.CD012551.pub3 PMC6494451 29757454

[6] Sönmez, N. C., Kiremit, M. C., Güney, S., Arisan, S., Akça, O., & Dalkılıç, A. (2011). Sexual dysfunction in type III chronic prostatitis (CP) and chronic pelvic pain syndrome (CPPS) observed in Turkish patients*. Int Urol Nephrol, 43*(2), 309–14. 10.1007/s11255-010-9809-5 20680450

[7] Leue, C., Kruimel, J., Vrijens, D., Masclee, A., van Os, J., & van Koeveringe, G. (2017). Functional urological disorders: a sensitized defence response in the bladder-gut-brain axis*. Nat Rev Urol, 14*(3), 153–163. 10.1038/nrurol.2016.227 27922040

[8] Ahn, S. G., Kim, S. H., Chung, K, I., Park, K. S., Cho, S. Y., & Kim, H. W. (2012). Depression, anxiety, stress perception, and coping strategies in korean military patients with chronic prostatitis/chronic pelvic pain syndrome*. Korean J Urol, 53*(9), 643–8. 10.4111/kju.2012.53.9.643 PMC3460008 23061003

[9] Lien, C. S., Chung, C. J., Lin, C. L., & Chang, C. H. (2020). Increased risk of prostatitis in male patients with depression*. World J Biol Psychiatry, 21*(2), 111–118. 10.1080/15622975.2018.1533994 31198079

[10] Fervaha, G., Izard, J. P., Tripp, D. A., Rajan, S., Leong, D. P., & Siemens, D. R. (2019). Depression and prostate cancer: a focused review for the clinician*. Urol Oncol, 37*(4), 282–288. 10.1016/j.urolonc.2018.12.020 30630735

[11] Porter, C. M., Shrestha, E., Peiffer, L. B., & Sfanos, K. S. (2018). The microbiome in prostate inflammation and prostate cancer*. Prostate Cancer Prostatic Dis, 21*(3), 345–354. 10.1038/s41391-018-0041-1 29795140

[12] Zhong, W., Wu, K., Long, Z., Zhou, X., Zhong, C., Wang, S., Lai, H., Guo, Y., Lv, D., Lu, J., & Mao, X. (2022). Gut dysbiosis promotes prostate cancer progression and docetaxel resistance via activating NF-κB-IL6-STAT3 axis*. Microbiome, 10*(1), 94. 10.1186/s40168-022-01289-w PMC9202177 35710492

[13] Bui, N. N., Li, C. Y., Wang, L. Y., Chen, Y. A., Kao, W. H., Chou, L. F., Hsieh, J. T., Lin, H., & Lai, C. H. (2023). Clostridium scindens metabolites trigger prostate cancer progression through androgen receptor signaling*. J Microbiol Immunol Infect, 56*(2), 246–256. 10.1016/j.jmii.2022.12.009 36639348

[14] An, J., Song, Y., Kim, S., Kong, H., & Kim, K. (2023). Alteration of gut microbes in benign prostatic hyperplasia model and finasteride treatment model*. Int J Mol Sci, 24*(6). 10.3390/ijms24065904 PMC10057928 36982979

[15] Mörkl, S., Butler, M. I., Holl, A., Cryan, J. F., & Dinan, T. G. (2020). Probiotics and the microbiota-gut-brain axis: focus on psychiatry*. Curr Nutr Rep, 9*(3), 171–182. 10.1007/s13668-020-00313-5 PMC7398953 32406013

[16] Huang, T. T., Lai, J. B., Du, Y. L., Xu, Y., Ruan, L. M., & Hu, S. H. (2019). Current understanding of gut microbiota in mood disorders: an update of human studies*. Front Genet, 10(*98). 10.3389/fgene.2019.00098 PMC6389720 30838027

[17] Du, H. X., Liu, Y., Zhang, L. G., Zhan, C. S., Chen, J., Zhang, M., Chen, X. G., Zhang, L., & Liang, C. Z. (2020). Abnormal gut microbiota composition is associated with experimental autoimmune prostatitis-induced depressive-like behaviors in mice*. Prostate, 80*(9), 663–673. 10.1002/pros.23978 32255522

[18] Kurokawa, S., Kishimoto, T., Mizuno, S., Masaoka, T., Naganuma, M., Liang, K. C., Kitazawa, M., Nakashima, M., Shindo, C., Suda, W., Hattori, M., Kanai, T., & Mimura, M. (2018). The effect of fecal microbiota transplantation on psychiatric symptoms among patients with irritable bowel syndrome, functional diarrhea and functional constipation: An open-label observational study*. J Affect Disord, 235*,506–512. 10.1016/j.jad.2018.04.038 29684865

[19] Chen, L., Zhang, M., & Liang, C. (2021). Chronic prostatitis and pelvic pain syndrome: another autoimmune disease? *Arch Immunol Ther Exp (Warsz), 69*(1), 24. 10.1007/s00005-021-00628-3 34523016

[20] Ma, L., Ni, Y., Wang, Z., Tu, W., Ni, L., Zhuge, F., Zheng, A., Hu, L., Zhao, Y., Zheng, L., & Fu, Z. (2020). Spermidine improves gut barrier integrity and gut microbiota function in diet-induced obese mice*. Gut Microbes, 12*(1), 1–19. 10.1080/19490976.2020.1832857 PMC7668533 33151120

[21] Caio, G., Lungaro, L., Segata, N., Guarino, M., Zoli, G., Volta, U., & De Giorgio, R. (2020). Effect of gluten-free diet on gut microbiota composition in patients with celiac disease and non-celiac gluten/wheat sensitivity*. Nutrients, 12*(6). 10.3390/nu12061832 PMC7353361 32575561

[22] Chen, J., Zhan, C., Zhang, L., Zhang, L., Liu, Y., Zhang, Y., Du, H., Liang, C., & Chen, X. (2019). The hypermethylation of Foxp3 promoter impairs the function of treg cells in EAP*. Inflammation, 42*(5), 1705–1718. 10.1007/s10753-019-01030-0 31209730

[23] Liu, F., Xu, X., Wang, Z., & Wu, P. (2022). Abnormal prostate microbiota composition is associated with experimental autoimmune prostatitis complicated with depression in rats*. Front Cell Infect Microbiol, 12*(966004. 10.3389/fcimb.2022.966004 PMC9563248 36250064

[24] Du, H. X., Liu, Y., Zhang, L. G., Zhan, C. S., Chen, J., Zhang, M., Chen, X. G., Zhang, L., & Liang, C. Z. (2020). Abnormal gut microbiota composition is associated with experimental autoimmune prostatitis‐induced depressive‐like behaviors in mice*. The Prostate, 80*(9), 663–673. 10.1002/pros.23978 32255522

[25] Gao, Y., Zhuang, Z., Lu, Y., Tao, T., Zhou, Y., Liu, G., Wang, H., Zhang, D., Wu, L., Dai, H., Li, W., & Hang, C. (2019). Curcumin mitigates neuro-inflammation by modulating microglia polarization through inhibiting TLR4 axis signaling pathway following experimental subarachnoid hemorrhage*. Front Neurosci, 13*(1223). 10.3389/fnins.2019.01223 PMC6872970 31803007

[26] Liu, H., Zhu, R., Liu, C., Ma, R., Wang, L., Chen, B., Li, L., Niu, J., Zhao, D., Mo, F., Fu, M., Bromme, D., Zhang, D., & Gao, S. (2017). Evaluation of decalcification techniques for rat femurs using HE and immunohistochemical staining*. Biomed Res Int, 2017*(9050754). 10.1155/2017/9050754 PMC5299168 28246608

[27] Kuniishi, H., Ichisaka, S., Yamamoto, M., Ikubo, N., Matsuda, S., Futora, E., Harada, R., Ishihara, K., & Hata, Y. (2017). Early deprivation increases high-leaning behavior, a novel anxiety-like behavior, in the open field test in rats*. Neurosci Res, 123*(27–35. 10.1016/j.neures.2017.04.012 28450152

[28] Yankelevitch-Yahav, R., Franko, M., Huly, A., & Doron, R. (2015). The forced swim test as a model of depressive-like behavior*. J Vis Exp,* 97. 10.3791/52587 PMC4401172 25867960

[29] Dang, R., Wang, M., Li, X., Wang, H., Liu, L., Wu, Q., Zhao, J., Ji, P., Zhong, L., Licinio, J., & Xie, P. (2022). Edaravone ameliorates depressive and anxiety-like behaviors via Sirt1/Nrf2/HO-1/Gpx4 pathway*. J Neuroinflammation, 19*(1), 41. 10.1186/s12974-022-02400-6 PMC8822843 35130906

[30] Fan, Y., Wang, J., He, N., & Feng, H. (2021). PLK2 protects retinal ganglion cells from oxidative stress by potentiating Nrf2 signaling via GSK-3β*. J Biochem Mol Toxicol, 35*(8), e22815. 10.1002/jbt.22815 34047419

[31] Tsunemori, H., & Sugimoto, M. (2021). Effects of inflammatory prostatitis on the development and progression of benign prostatic hyperplasia: A literature review*. Int J Urol, 28*(11), 1086–1092. 10.1111/iju.14644 34342061

[32] Nikolova, V. L., Smith, M. R. B., Hall, L. J., Cleare, A. J., Stone, J. M., & Young, A. H. (2021). Perturbations in Gut Microbiota Composition in Psychiatric Disorders: A Review and Meta-analysis*. JAMA Psychiatry, 78*(12), 1343–1354. 10.1001/jamapsychiatry.2021.2573 PMC8444066 34524405

[33] Eyupoglu, N. D., Ergunay, K., Acikgoz, A., Akyon, Y., Yilmaz, E., & Yildiz, B. O. (2020). Gut Microbiota and Oral Contraceptive Use in Overweight and Obese Patients with Polycystic Ovary Syndrome*. J Clin Endocrinol Metab, 105*(12). 10.1210/clinem/dgaa600 32860695

[34] Zhang, C., Zhang, W., Zhang, J., Jing, Y., Yang, M., Du, L., Gao, F., Gong, H., Chen, L., Li, J., Liu, H., Qin, C., Jia, Y., Qiao, J., Wei, B., Yu, Y., Zhou, H., Liu, Z., Yang, D., & Li, J. (2018). Gut microbiota dysbiosis in male patients with chronic traumatic complete spinal cord injury*. J Transl Med, 16*(1), 353. 10.1186/s12967-018-1735-9 PMC6293533 30545398

[35] Shoskes, D. A., Altemus, J., Polackwich, A. S., Tucky, B., Wang, H., & Eng, C. (2016). The urinary microbiome differs significantly between patients with chronic prostatitis/chronic pelvic pain syndrome and controls as well as between patients with different clinical phenotypes*. Urology, 92*(26–32). 10.1016/j.urology.2016.02.043 26970449

[36] Hou, Y. F., Shan, C., Zhuang, S. Y., Zhuang, Q. Q., Ghosh, A., Zhu, K. C., Kong, X. K., Wang, S. M., Gong, Y. L., Yang, Y. Y., Tao, B., Sun, L. H., Zhao, H. Y., Guo, X. Z., Wang, W. Q., Ning, G., Gu, Y. Y., Li, S. T., & Liu, J. M. (2021). Gut microbiota-derived propionate mediates the neuroprotective effect of osteocalcin in a mouse model of Parkinson's disease*. Microbiome, 9*(1), 34. 10.1186/s40168-020-00988-6 PMC7849090 33517890

[37] Van Ameringen, M., Turna, J., Patterson, B., Pipe, A., Mao, R. Q., Anglin, R., & Surette, M. G. (2019). The gut microbiome in psychiatry: A primer for clinicians*. Depress Anxiety, 36*(11), 1004–1025. 10.1002/da.22936 31356715

[38] Megur, A., Baltriukienė, D., Bukelskienė, V., & Burokas, A. (2020). The microbiota-gut-brain axis and alzheimer's disease: neuroinflammation is to blame? *Nutrients, 13*(1). 10.3390/nu13010037 PMC7824474 33374235

[39] Caputi, V., & Giron, M. C. (2018). Microbiome-gut-brain axis and toll-like receptors in parkinson's disease*. Int J Mol Sci, 19*(6). 10.3390/ijms19061689 PMC6032048 29882798

[40] Liang, S., Wu, X., Hu, X., Wang, T., & Jin, F. (2018). Recognizing depression from the microbiota-gut-brain axis*. Int J Mol Sci, 19*(6). 10.3390/ijms19061592 PMC6032096 29843470

[41] Cohen, L. J., Esterhazy, D., Kim, S. H., Lemetre, C., Aguilar, R. R., Gordon, E. A., Pickard, A. J., Cross, J. R., Emiliano, A. B., Han, S. M., Chu, J., Vila-Farres, X., Kaplitt, J., Rogoz, A., Calle, P. Y., Hunter, C., Bitok, J. K., & Brady, S. F. (2017). Commensal bacteria make GPCR ligands that mimic human signalling molecules*. Nature, 549*(7670), 48–53. 10.1038/nature23874 PMC5777231 28854168

[42] Tognini, P. (2017). Gut Microbiota: A potential regulator of neurodevelopment*. Front Cell Neurosci, 11*(25. 10.3389/fncel.2017.00025 PMC5293830 28223922

[43] De Palma, G., Lynch, M. D., Lu, J., Dang, V. T., Deng, Y., Jury, J., Umeh, G., Miranda, P. M., Pigrau Pastor, M., Sidani, S., Pinto-Sanchez, M. I., Philip, V., McLean, P. G., Hagelsieb, M. G., Surette, M. G., Bergonzelli, G. E., Verdu, E. F., Britz-McKibbin, P., Neufeld, J. D., Collins, S. M., & Bercik, P. (2017). Transplantation of fecal microbiota from patients with irritable bowel syndrome alters gut function and behavior in recipient mice*. Sci Transl Med, 9*(379). 10.1126/scitranslmed.aaf6397 28251905

[44] Chevalier, G., Siopi, E., Guenin-Macé, L., Pascal, M., Laval, T., Rifflet, A., Boneca, I. G., Demangel, C., Colsch, B., Pruvost, A., Chu-Van, E., Messager, A., Leulier, F., Lepousez, G., Eberl, G., & Lledo, P. M. (2020). Effect of gut microbiota on depressive-like behaviors in mice is mediated by the endocannabinoid system*. Nat Commun, 11*(1), 6363. 10.1038/s41467-020-19931-2 PMC7732982 33311466

[45] He, J., Xu, S., Zhang, B., Xiao, C., Chen, Z., Si, F., Fu, J., Lin, X., Zheng, G., Yu, G., & Chen, J. (2020). Gut microbiota and metabolite alterations associated with reduced bone mineral density or bone metabolic indexes in postmenopausal osteoporosis*. Aging (Albany NY), 12*(9), 8583–8604. 10.18632/aging.103168 PMC7244073 32392181

[46] Luo, D., Chen, K., Li, J., Fang, Z., Pang, H., Yin, Y., Rong, X., & Guo, J. (2020). Gut microbiota combined with metabolomics reveals the metabolic profile of the normal aging process and the anti-aging effect of FuFang Zhenshu TiaoZhi(FTZ) in mice*. Biomed Pharmacother, 121*(109550. 10.1016/j.biopha.2019.109550 31704617

[47] Su, X., Gao, Y., & Yang, R. (2022). Gut microbiota-derived tryptophan metabolites maintain gut and systemic homeostasis*. Cells, 11*(15). 10.3390/cells11152296 PMC9330295 35892593

[48] Chang, L., Wei, Y., & Hashimoto, K. (2022). Brain-gut-microbiota axis in depression: a historical overview and future directions*. Brain Res Bull, 182,*44–56. 10.1016/j.brainresbull.2022.02.004 35151796

[49] Soultanov, V. S. (2016). New hepatic and neurological ckinical implications of long-chain plant polyprenols acting on the mammalian isoprenoid pathway*. Eksp Klin Gastroenterol, 11*, 104–113. 29889454

[50] Sabelli, H. C., Fawcett, J., Gusovsky, F., Javaid, J., Edwards, J., & Jeffriess, H. (1983). Urinary phenyl acetate: a diagnostic test for depression? *Science, 220*(4602), 1187–8. 10.1126/science.6857245 6857245

