# Supplementary material for: Abnormal Gut Microbiota is Associated with Depressive-like Behavior in the Rat Model of Autoimmune Prostatitis
Source: Appl Biochem Biotechnol. 2025 Jun 27;197(9):5629–48. doi: 10.1007/s12010-025-05294-1 (PMC12568870; doi:10.1007/s12010-025-05294-1)
Supplement: Supplementary file 1 — (DOCX 16.4 KB) [file 12010_2025_5294_MOESM1_ESM.docx]

**Supplement Table S1** **List of reagents used in the study**

| **Reagent** | **Vendor** | **Catalog Number** |
| --- | --- | --- |
| TritonX-100 | G-Biosciences | 786-513 |
| protease inhibitor | Sigma-Aldrich | P8340 |
| BCA kit | Abcam | ab102536 |
| Freund's adjuvant | Sigma-Aldrich | F5581 |
| 0.9% saline | MOLTOX | 22-143.020I |
| penicillin | Sigma-Aldrich | 19532 |
| paraformaldehyde (PFA) | Sigma-Aldrich | 30525-89-4 |
| TNF-α ELISA kit | Shanghai Xitang Biotechnology Co., Ltd, | F16960 |
| IL-6 ELISA kit | Shanghai Xitang Biotechnology Co., Ltd, | F3743 |
| IL-1β ELISA kit | Shanghai Xitang Biotechnology Co., Ltd, | F3739 |
| TRIzol | Invitrogen | 15596026 |
| PrimeScriptRT reagent Kit | Takara | RR047A |
| Power up SYBR Green | ABI | A25779 |
| HE staining buffer | BASO | BA4025 |
